# Musashi2 as a novel predictive biomarker for liver metastasis and poor prognosis in colorectal cancer

**DOI:** 10.1002/cam4.624

**Published:** 2016-01-18

**Authors:** Zhen Zong, Taicheng Zhou, Liangjun Rao, Zhipeng Jiang, Yingru Li, Zehui Hou, Bin Yang, Fanghai Han, Shuang Chen

**Affiliations:** ^1^ Department of Gastroenterological SurgerySun Yat‐sen Memorial hospitalSun Yat‐sen UniversityGuangzhouChina; ^2^ Departments of Gastroenterological Surgery and Hernia CenterThe Sixth Affiliated HospitalSun Yat‐sen UniversityGuangzhouChina; ^3^ Department of Gastroenterological SurgeryGuangzhou First People's HospitalGuangzhou Medical UniversityGuangzhou Digestive Disease CenterGuangzhouChina; ^4^ Department of RadiologyThe First Affiliated HospitalSun Yat‐sen UniversityGuangzhouChina

**Keywords:** Colorectal cancer, metastasis, musashi2, prognosis

## Abstract

Aberrant expression of musashi2 (MSI‐2) has been detected in several malignancies. However, its role in the progression of colorectal cancer (CRC) remains unknown. Our study was designed to investigate the expression and prognostic significance of MSI‐2 protein in patients with colorectal cancer. The expression of MSI‐2 was detected in 164 patients’ colorectal cancer and control specimens by the tissue microarray technique and immunohistochemical staining. The correlations between MSI‐2 expression and clinicopathological variables including overall survival were analyzed. The prognostic value of liver metastasis is evaluated by logistic regression and receiver operating characteristic (ROC) analysis. MSI‐2 was highly expressed in 32.9% (54/164) of the colorectal cancer. Overexpression of MSI‐2 was associated with depth of invasion, lymph node metastasis, distant metastasis, liver metastasis, Tumor Node Metastasis (TNM) clinical stage, and Carcinoembryonicantigen (CEA) level (*P *= 0.040, 0.014, <0.001, <0.001, 0.003, and 0.002, respectively). In the Cox multivariate test, MSI‐2 overexpression, lymph node metastasis, and distant metastasis were found to be the independent prognostic factors (*P* = 0.027, 0.010, and 0.001, respectively). Further logistic regression suggested that TNM stage and MSI‐2 high expression were related to liver metastasis in colorectal cancer patients. Conclusively, our study indicates that MSI‐2 overexpression is associated with an unfavorable prognosis and may be a potential biomarker for liver metastasis in colorectal cancer patients.

## Introduction

Colorectal cancer (CRC) is one of the most common malignant tumors associated with high recurrence incidence and poor prognosis, especially in the developed countries 1, 2. Although the development of earlier diagnosis and comprehensive treatment is dramatic, the mortality rate of colorectal cancer is still occupying the third place in both male and female patients 1, 3. The main reason is the local recurrence and distant metastasis occupying a large proportion of CRC patient population. The liver is the most common site of distant metastatic disease, leading to shorter survival of the patients 4, 5. Synchronous liver metastasis accounted for approximately 15–25% of all CRC patients at the time of initial diagnosis 6. Meantime, metachronous liver metastasis amounted to 10–25% of CRC patients even after curative resection of the primary lesion 7. The exact mechanism responsible for the proliferation and metastasis of the colorectal cancer is still unclear 8. Therefore, it is imperative to identify the predictive markers associated with the colorectal cancer progression and invasiveness, which is helpful for patients to select suitable therapeutic regimens and regular surveillance.

The musashi (MSI) family of RNA‐binding proteins acts as a posttranslational repressor of target mRNA 9, including two MSI homologs, MSI‐1 and MSI‐2 10. The human MSI‐2 gene is located on 17q22 chromosome 11, 12, encoding an RNA‐binding protein distributed in the stem cell compartment of neural 13, hematopoietic 14, pancreatic 15, and epithelial tissues 16, 17. MSI‐2 plays an important role in regulating proliferation and differentiation of the nervous 18, 19 and hematopoietic systems 20. In addition to influencing the development of the stem cells, MSI‐2 gene has also been linked to tumorigenesis. Importantly, emerging evidence shows that MSI‐2 is overexpressed in malignancies 21, 22, 23. It is an indicator of poor prognosis and relapse in chronic myelogenous leukemia (CML) 9, acute myeloid leukemia (AML)^22^, ^23^, and hepatocellular carcinoma 17. Recent findings identify MSI2 as a central component in an unappreciated oncogenic pathway promoting intestinal transformation via the PDK–AKT–mTORC1 axis 24. However, the clinicopathologic significance and mechanism of MSI‐2 involvement in the aggression of the colorectal cancer is not completely understood. In this study, we profiled the expression status of MSI‐2 in the colorectal cancer and evaluated the prognostic significance of MSI‐2 in liver metastasis of colorectal cancer. To our knowledge, this was first study designed to investigate the prognostic and predictive significance of intratumoral MSI‐2 expression in the colorectal cancer.

## Material and Method

### Patients and tissue specimens

Colorectal cancer specimens (tumor and paired adjacent nontumorous tissues) were obtained from 164 patients with histologically confirmed who underwent colon or rectum resection at the Sun Yat‐sen Memorial Hospital of Sun Yat‐sen University from December 2007 to December 2012. None of the patients received any preoperative chemotherapy or radiation, and these CRC patients were monitored after surgery until 31 March 2015. Detailed clinicopathological parameters are listed in Table 1. The overall survival (OS) was calculated from the day of surgery to the date of death or the last follow‐up. Ethical approval for human subjects was obtained from the ethics committee of Sun Yat‐sen Memorial Hospital.

**Table 1:** Correlation between intratumoral MSI‐2 protein expression and clinicopathological parameters in patients with colorectal cancer (CRC)

Variables	Patients		MSI‐2 expression		*P* value
	No., 164	%, 100%	High, 54	Low, 110	
Age (years)					0.510
≤60	76	46.3	27	49	
>60	88	53.7	27	61	
Gender					0.537
Male	107	65.2	37	70	
Female	57	34.8	17	40	
Tumor location					0.326
Colon	110	67.1	39	71	
Rectum	54	32.9	15	39	
Tumor size					0.061
≤5 cm	132	80.5	39	93	
>5 cm	32	19.5	15	17	
Gross appearance					0.512
Exophytic	67	40.9	24	43	
Ulcerative	97	59.1	30	65	
Histological type					0.125
Adenocarcinoma	143	87.2	44	99	
Mucinous adenocarcinoma	21	12.8	10	11	
Tumor differentiation					0.138
Well, moderate	124	75.6	37	87	
Poor and others	40	24.4	17	23	
Depth of invasion					**0.040**
T1	7	4.3	0	7	
T2	22	13.4	7	15	
T3	122	74.4	39	83	
T4	13	7.9	8	5	
Lymph node metastasis					**0.014**
N0	96	58.5	27	69	
N1	42	25.6	12	30	
N2	26	15.9	15	11	
Distant metastasis					**<0.001**
M0	145	88.4	41	104	
M1	19	11.6	13	6	
Liver metastasis					**0.005**
Not	130	79.3	36	94	
Yes	34	20.7	18	16	
TNM stage					**0.003**
I	20	12.2	3	17	
II	71	43.3	19	52	
III	55	33.5	20	35	
IV	18	11.0	12	6	
CEA					**0.002**
≤5 ng/mL	101	61.6	24	77	
>5 ng/mL	63	38.4	30	33	

Bold values (*P < *0.05) are statistically significant.

### Tissue microarray and immunohistochemistry

Formalin‐fixed, paraffin‐embedded tissue specimens were cut into 4‐*μ*m sections. Tissue blocks were constructed in a new recipient paraffin tissue array block using a commercially available microarray instrument (Sliver Spring, Inc, Montgomery, Maryland, USA). Slices of 4 *μ*m in thickness were cut from each tissue microarray paraffin blocks mounted on positively charged slides and stained with hematoxylin and eosin. The specimens were deparaffinized in xylene and rehydrated using a series of graded alcohols after being dried at 62°C for 2 h. The sections were heated at 60°C for 15 min, de‐waxed in xylene, and dehydrated in graded ethanol to distilled water. The slides were retrieved in 0.01 mol/L citric acid buffer (PH 6.0) using a microwave oven for 15 min; 3% hydrogen peroxide in methanol was used to block endogenous peroxidase and nonspecific staining for 15 min. The sections were incubated with a rabbit polyclonal antibody against human MSI‐2 (Abcam Biotechnology, Inc, Shanghai, China) overnight at 4°C. Horseradish peroxidase (HPR)‐labeled goat anti‐rabbit IgG secondary antibody (SantaCruz Biotechnology, Inc, Shanghai, China) stayed for 10 min at 37°C. Finally, the sections were visualized using diaminobenzidine (Zhongshan Golden Bridge, Inc, Beijing, China) and counterstain with hematoxylin (Zhongshan Golden Bridge, Inc). Negative controls were treated identically, just without the primary antibody.

### Evaluations of immunohistochemical findings

Two researchers who were blinded to patients’ outcome evaluated immunoreactivity independently. The intensity of the Immunohistochemistry (IHC) staining of MSI‐2 was evaluated using the semiquantitative scoring system. Staining intensity for MSI‐2 was scored from 0 to 3. Staining extent was scored from 0% to 100%. A composite score was obtained by multiply the intensity by the extent. The score ≤1.5 was considered low expression, and the score between 1.5 and 3 was considered high expression.

### Statistical analysis

All data were analyzed using SPSS statistic software (Version 20, IBM, Inc, Armonk, New York, USA). The relationships between clinicopathological parameters and MSI‐2 expression were analyzed using the chi‐square test or Fisher's extract test. Survival curves were constructed using the Kaplan–Meier method and analyzed by the log‐rank test. The Kaplan–Meier method was used to analyze colorectal cancer patients’ overall survival (OS). The log‐rank test was used to analyze survival differences. A Cox proportional hazards model with forward stepwise selection was used to calculate univariate and multivariate hazard ratio for the study variables. The prognostic value of liver metastasis was evaluated by logistic regression. Receiver operating characteristic (ROC) analysis was used to compare the sensitivity and specificity for the prediction of liver metastasis in CRC patients. A *P* value of <0.05 was considered statistically significance.

## Results

### The protein level of MSI‐2 was unregulated in CRC tissues

**Figure 1:**
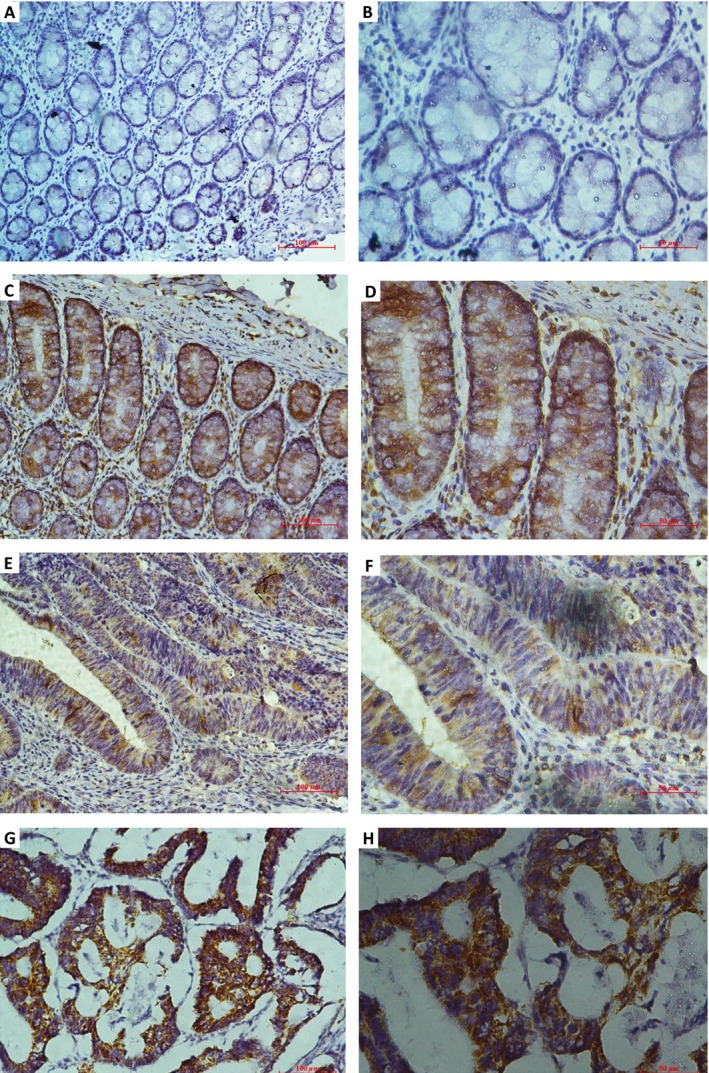
Expression of musashi2 (MSI‐2) in 164 paired colorectal cancer and adjacent nontumorous tissues by immunohistochemical staining. (A and B) Low MSI‐2 expression in the normal samples; (C and D) high MSI‐2 expression in the normal samples; (E and F) low MSI‐2 expression in the colorectal cancer tissues; (G and H) high MSI‐2 expression in the colorectal cancer tissues (original magnification in A, C, E, and G, 200×; original magnification in B, D, F, and H, 400×).

We detected MSI‐2 expression by immunohistochemical analyses in a tissue microarray, containing 164 cases of primary colorectal cancer paired with nontumorous specimens. MSI‐2 protein was elevated in CRC tissues and mainly localized in the cytoplasm of the cancer cells. The intensity of the immunohistochemical staining was variable (Fig.**** 1). According to the previous criterion discussed in Material and Method section 17, approximately 32.9% (54/164) of primary cancer lesions was scored as high MSI‐2 expression when compared with adjacent noncancerous tissue.

### The relationship between MSI‐2 expression and clinicopathological factors in CRC patients

Immunohistochemical staining of MSI‐2 levels was statistically analyzed to determine the relationship with critical clinicopathological variables among the colorectal cancer patients. As shown in Table 1, intratumoral MSI‐2 showed a positive correlation with depth of invasion (*P* = 0.040), lymph node metastasis (*P* = 0.014), distant metastasis (*P < *0.001), synchronous and metachronous liver metastasis (*P* < 0.001), TNM clinical stage (*P* = 0.003), and CEA level (*P* = 0.002). There were no significant differences in age, gender, tumor location and size, gross appearance, and histological type and tumor differentiation between the high and low intratumoral MSI‐2 expression groups.

### Association of MSI‐2 expression with overall survival of CRC patients

**Figure 2:**
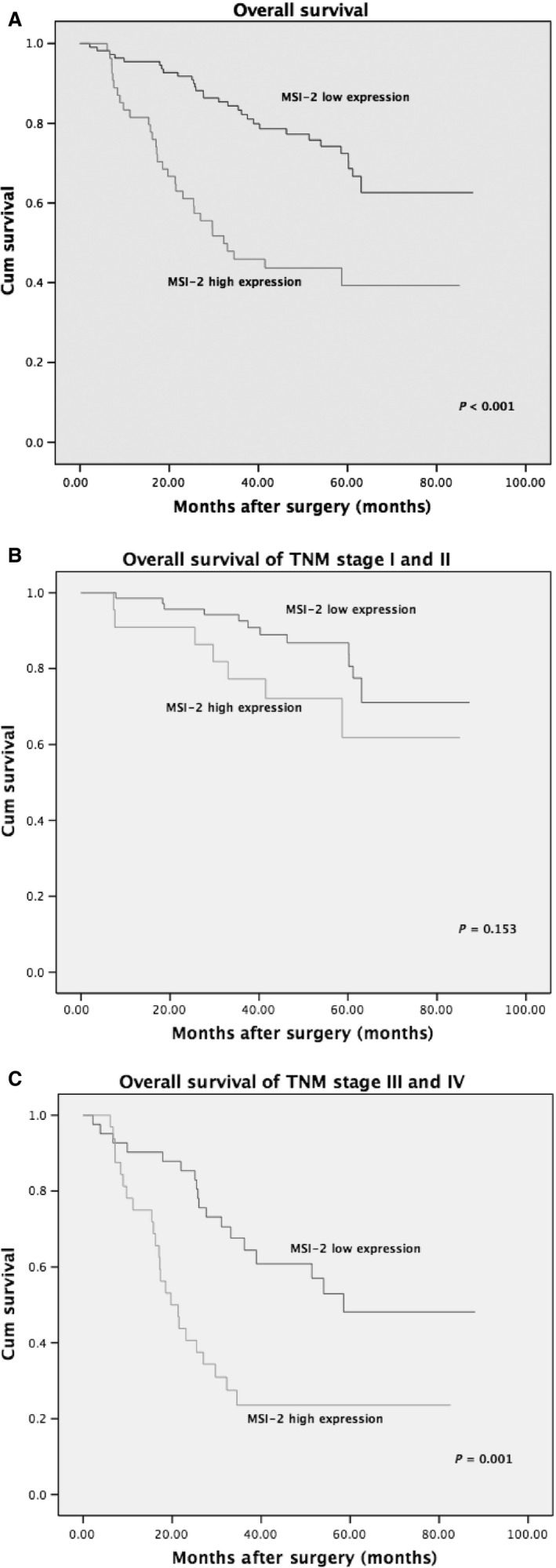
Kaplan–Merier survival curve of overall survival according to MSI‐2 expression in colorectal cancer patients: (A) in the whole study patients; (B) in the stage I and II patients; and (C) in the stage III and IV patients.

According to the intratumoral MSI‐2 expression, we compared the overall survival (OS) to further investigate the prognostic values of MSI‐2 expression in CRC patients. As shown in Figure**** 2, in the group combining stage I, II, III, and IV patients, the Kaplan–Meier survival analysis revealed that the overall survival of CRC patients with MSI‐2 high expression was significantly poorer than those patients with MSI‐2 low expression (log‐rank test, *P* < 0.001). In stage I and II patients, overall survival rate of patients with MSI‐2 high expression did not significantly differ from that of patients with MSI‐2 low expression (log‐rank test, *P* = 0.153). However, in the group combining stage III and IV patients, overall survival rate of patients with MSI‐2 high expression was significantly lower compared with patients who had MSI‐2 low expression (log‐rank test, *P* = 0.001).

Univariate analyses were performed to estimate the clinical significance of various prognostic factors that might influence overall survival in 164 patients with CRC. Collectively, histological type, tumor differentiation, lymph node and distant metastasis, synchronous and metachronous liver metastasis, TNM stage, CEA level, and MSI‐2 overexpression were associated with colorectal cancer (CRC)‐specific survival in the study population (*P* = 0.034, 0.036, <0.001, <0.001, <0.001, <0.001, 0.009, and<0.001, respectively, log‐rank test, shown in Table 2).

**Table 2:** Univariate analysis of clinicopathological factors for OS in 164 colorectal cancer (CRC) patients

Characteristics	Overall Survival (OS)		
	Hazard ratio	95% CI	*P* value
Age:>60 versus ≤60	0.856	0.520,1.409	0.541
Gender: female versus male	1.306	0.783,2.178	0.306
Tumor location: rectum versus colon	1.437	0.864,2.390	0.162
Tumor size:>5 versus ≤5 cm	1.270	0.700,2.306	0.432
Gross appearance: ulcerative versus exophytic	1.606	0.942,2.738	0.082
Histological type: mucinous adenocarcinoma versus adenocarcinoma	1.981	1.054,3.726	**0.034**
Tumor differentiation: poor and others versus well, moderate	1.772	1.038,3.023	**0.036**
T:T3, T4 versus T1, T2	2.255	0.971,5.235	0.059
Lymph node metastasis: positive versus negative	2.740	1.649,4.553	**<0.001**
Distant metastasis: M1 versus M0	5.479	3.065,9.794	**<0.001**
TNM Stage: III+IV versus I+II	3.994	2.335,6.834	**<0.001**
Liver metastasis: positive versus negative	5.362	3.234,8.889	**<0.001**
CEA:>5 versus ≤5 ng/mL	1.947	1.180,3.210	**0.009**
MSI‐2 expression: high versus low	2.888	1.750,4.766	**<0.001**

Bold values (*P < *0.05) are statistically significant.

CI, confidence interval.

In order to evaluate the robustness of the prognostic value of intratumoral MSI‐2 expression, the Cox multivariate regression test was performed to select independent risk factors, which had significance in univariate analyses. Intratumoral MSI‐2 expression (hazard ratio [HR], 1.894; 95% confidence interval [CI], 1.076–3.334; *P* = 0.027), lymph node metastasis (HR, 2.091; 95% CI, 1.195–3.659; *P* = 0.010), and distant metastasis (HR, 3.048; 95% CI, 1.597–5.820; *P* = 0.001) were shown in Table 3. These three characteristics were recognized as independent prognostic factors for overall survival (OS) in this study population. Taken together, our findings indicate that MSI‐2 cytoplasm high expression is an independent poor prognostic marker in colorectal cancer and a useful marker to predict the survival of patients with CRC.

**Table 3:** Multivariate Cox regression model for OS in 164 colorectal cancer (CRC) patients

Characteristics	Overall Survival (OS)		
	Hazard ratio	95% CI	*P* value
Histological type: mucinous adenocarcinoma versus adenocarcinoma	1.003	0.459,2.194	0.993
Tumor differentiation: poor and others versus well, moderate	1.138	0.577,2.245	0.710
Lymph node metastasis: positive versus negative	2.091	1.195,3.659	**0.010**
Distant metastasis: M1 versus M0	3.048	1.597,5.820	**0.001**
CEA: >5 versus ≤5 ng/mL	1.583	0.938,2.669	0.085
MSI‐2 expression: high versus low	1.894	1.076,3.334	**0.027**

Bold values (*P < *0.05) are statistically significant.

CI, confidence interval.

### Logistic regression and receiver operating characteristic analysis were used to predict liver metastasis of colorectal cancer patients

In a total of 164 enrolled cases, 34 cases involved synchronous and metachronous liver metastasis. We further investigated the correlation between intratumoral MSI2 expression and distant liver metastasis of colorectal cancer. Cases with MSI‐2 high expression exhibited a significantly higher rate of synchronous and metachronous liver metastasis than those MSI‐2 low expressions did. As shown in Table 4, MSI‐2 protein expression and TNM stage were significantly related to the occurrence of synchronous and metachronous liver metastasis (*P* = 0.029 and 0.012, respectively).

**Table 4:** Logistic regression analysis of the factors related to liver metastasis of colorectal cancer (CRC) patients

Characteristics	OR	95% CI	*P* value
Stage: III+IV versus I+II	2.873	1.265,6.525	**0.012**
MSI‐2 expression: high versus low	2.439	1.095,5.432	**0.029**
Constant	0.105		

Bold values (*P < *0.05) are statistically significant.

CI confidence interval; OR, odd rate.

**Figure 3:**
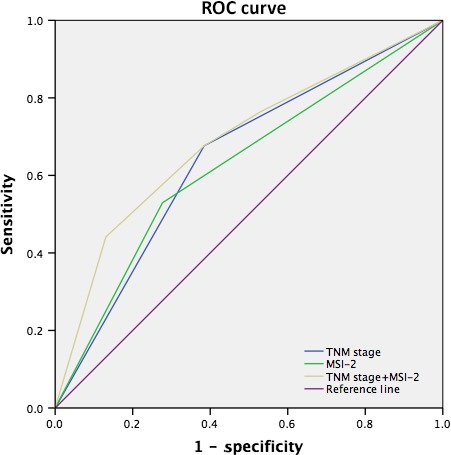
Receiver operating characteristic (ROC) analyses for the prediction of liver metastasis in colorectal cancer patients.

A molecular prognostic stratification scheme incorporating MSI‐2 expression was determined by using ROC analysis. ROC analyses of the sensitivity and specificity for the prediction of liver metastasis were shown by the combined MSI‐2 expression and TNM stage model, the TNM stage model alone, and the MSI‐2 expression model alone. The combination of intratumoral MSI‐2 expression and TNM stage (Area under curve [AUC] [95% CI], 0.69 [0.584–0.796]) showed a better predictive value than the TNM stage (AUC [95% CI], 0.646[0.543–0.749]) or intratumoral MSI‐2 expression (AUC [95% CI], 0.626 [0.517–0.735]) alone (Fig.3).

## Discussion

Because of the high incidence of localize recurrence and distant metastasis, the long‐term survival rate of colorectal cancer patients remains unsatisfactory. Therefore, the early detection of new predictive biomarkers of colorectal cancer prognosis is critical. Based on our data, we would speculate that increased intratumoral MSI‐2 expression could be one of these kinds of prognostic markers in colorectal cancer.

Musashi gene expression was reported to be closely associated with poor prognosis in cases of breast cancer 25, gallbladder adenocarcinoma 26, leukemia 9, 22, 23, and other. Two members of musashi gene family (MSI‐1 and MSI‐2) exhibit a high degree of sequence similarity 13. MSI‐1 protein has also found to be highly expressed in colorectal cancer, and its elevated expression is associated with poor overall survival (OS) and metastasis‐free survival (MFS) 27.

MSI‐2 is preferentially expressed in the hematopoietic 23 and neural system 13. Previous studies suggested that MSI‐2 functions as a tumor promoter that is overexpressed in these types of cancers. As our knowledge, MSI‐2 could inhibit the expression of NumB 9, 28, a well‐accepted inhibitor of the Notch signaling pathway, thereby activate the Notch signal pathway 29, 30, 31. Recent studies showed that MSI‐2 still plays an important role in the carcinogenesis and metastasis of some other solid tumors 15, 32, 33, 34. Aberrant overexpression of MSI‐2 was found in hepatic carcinoma 17. MSI‐2 enhances hepatocellular carcinoma (HCC) invasion and metastasis by driving epithelial–mesenchymal transition (EMT). MSI‐2 has emerged as a regulator of EMT‐like transformation in hepatic tumors.

However, the precise function of MSI‐2 in colorectal cancer remains unclear. MSI‐2 may also be involved in pathways that promote cancer invasion and metastasis, such as the PDK–AKT–mTORC1 pathway, which is responsible for clinical aggressiveness and poor prognosis in colorectal cancer. Wang demonstrated that MSI‐2 is an important component, which promotes intestinal transformation via the PDK–AKT–mTORC1 axis 24. Their RNASeq data indicated that MSI‐2 acts as a pleiotropic inhibitor of known intestinal tumor suppressors, including Lrig1, Bmpr1a, Cdkn1a, and Pten. Interestingly, Katz found that MSI‐2 gene is associated with an epithelial‐luminal cell state and MSI‐2 protein regulates translation of genes implicated in epithelial to mesenchymal transition (EMT) in neural and mammary cell types 35. Further studies are needed to investigate the potential mechanism in colorectal cancer.

Our data provided the evidence that MSI‐2 expression was also linearly correlated with advanced clinicopathological factors. This finding was consistent with the previous research results of MSI‐2 in HCC 17. MSI‐2 expression levels may be useful for the establishment of rational treatment selection criteria after colectomy. According to TNM stage, colorectal cancer patients in this study were stratified into two groups: stage I, II and stage III, IV. In this study, we confirmed that there was a statistical difference in survival for patients with stage III and IV colorectal cancer, while patients with stage I and II do not receive significant difference. Thus, a more precise prognostic value of MSI‐2 expression is shown, which bring a step closer toward personalized therapeutic.

Moreover, our results imply that cytoplasm MSI‐2 overexpression might be a biomarker for screening patients with colorectal cancer to have the risk of liver metastasis. The unadjusted odd rate (OR) of MSI‐2 for the risk of liver metastasis was 2.873 (95% CI, 1.265–6.525). Thus, MSI‐2 high expression has the potential utility to serve as a novel predictive biomarker of liver metastasis.

The AJCC TNM classification is an internationally used system, which provides prognostic information and predictive value for liver metastasis in colorectal patients. In this study, we developed a new predictive indicator system using integration of intratumoral MSI‐2 expression into the current TNM stage system. Another interesting point found from this study is that the combination system improves the predictive value of liver metastasis over the single one (AUC = 0.69, 0.646, and 0.626, respectively, *P* = 0.001). It is helpful to identify subsets of colorectal cancer patients with much higher risk of liver metastasis after surgery. The patients with MSI‐2 high expression might require strict surveillance or adjuvant chemotherapy.

In addition, our data provide some insights into the roles of MSI‐2 in colorectal cancer, and warrant further investigation into the specific functions of MSI‐2 protein, which is likely to find a new therapeutic target for colorectal cancer.

A limitation of this study is that tissue microarrays may not be truly representative of the entire tumor. This limitation can be overcome by using multiple punches of every sample on the microarray. To our knowledge, we present the first report that MSI‐2 overexpression is an independent poor prognostic for overall survival following resection of CRC patients. More investigations are needed to confirm this conclusion in the future.

In summary, we provide the convincing evidence for the first time that the expression of cytoplasm MSI‐2 is unregulated in colorectal cancer patients. Cytoplasm MSI‐2 overexpression in patients is associated with an unfavorable prognosis and may be a potential predictive biomarker for liver metastasis in colorectal cancer patients.

## Conflict of Interest

None declared.
